# Accelerometer-based 24-h movement behaviours in children and adolescents with Down syndrome

**DOI:** 10.1007/s00431-026-07507-z

**Published:** 2026-10-12

**Authors:** Valeria Calcaterra, Matteo Vandoni, Alessandro Gatti, Paolo Maugeri, Alessandra Gazzarri, Jonabel Dolor, Anna Odone, Maddalena Gaeta, Gianvincenzo Zuccotti

**Affiliations:** 1 Pediatric and Adolescent Unit, Department of Internal Medicine and Therapeutics, University of Pavia, Pavia, Italy; 2 Department of Pediatrics, Buzzi Children’s Hospital, Milano, Italy; 3 Laboratory of Adapted Motor Activity (LAMA), Department of Public Health, Experimental Medicine and Forensic Science, University of Pavia, Pavia, Italy; 4 National PhD Programme in One Health Approaches to Infectious Diseases and Life Science Research, Department of Public Health, Experimental and Forensic Medicine, University of Pavia, Pavia, Italy; 5 Associazione Vivi Down Onlus, Milan, Italy; 6 Department of Public Health, Experimental and Forensic Medicine, University of Pavia, Pavia, Italy; 7 Medical Direction, Fondazione IRCCS Policlinico San Matteo, Pavia, Italy; 8 Department of Biomedical and Clinical Science, University of Milano, Milan, Italy

**Keywords:** Down syndrome, Physical activity, Sedentary behaviour, Sleep, Accelerometry

## Abstract

Children and adolescents with Down syndrome (DS) may show altered 24-h movement behaviours. This study compared physical activity, sedentary behaviour, sleep and rest–activity rhythms in paediatric DS with those of peers with normal weight and obesity. This cross-sectional study included 51 participants aged 8–18 years: 17 with DS, 17 with normal weight and 17 with obesity. Seven-day accelerometry assessed activity, sedentary behaviour, sleep and rest–activity rhythms. Group differences were analysed using adjusted ANCOVA, PERMANOVA and principal component analysis. Compared with normal-weight peers, participants with DS had significantly greater sedentary time and lower light, moderate and moderate-to-vigorous physical activity (all *p* < 0.001). They also exhibited longer sedentary bouts (*p* < 0.001), shorter physical-activity bouts (*p* = 0.037), a lower probability of transitioning from sedentary time to physical activity (*p* < 0.001) and a higher probability of transitioning back to sedentary time (*p* = 0.027). Rest–activity rhythms were less stable, with lower interdaily stability (*p* = 0.030), greater intradaily variability (*p* = 0.008), lower M5 activity (*p* < 0.001) and later M5 timing (*p* = 0.015) and cosinor acrotime (*p* < 0.001). Most activity and rhythmicity outcomes were similar in the DS and obesity groups. Participants with DS slept longer but had poorer sleep continuity. Overall 24-h behavioural profiles differed significantly among groups (*p* < 0.001).

*Conclusion*: Children and adolescents with DS display a sedentary, low-movement and less stable 24-h behavioural profile resembling that observed in obesity. Integrated assessment of activity, sedentary accumulation, sleep continuity and rhythmicity may support early identification of modifiable risk and guide individualized, family-centred interventions.

**Table:** 

**What is Known:** • *Youth with Down syndrome are generally less active and more sedentary than typically developing peers and frequently experience disrupted sleep.* • *Objective studies integrating activity, sedentary accumulation, sleep and rest–activity rhythms across 24 h remain limited.*	
**What is New:** • *Youth with Down syndrome showed a sedentary, low-movement, fragmented and less stable 24-h profile resembling that of peers with obesity.* • *Longer sleep did not indicate better sleep, as nocturnal wakefulness remained high and sleep efficiency was low.*

**Supplementary information:**

The online version contains supplementary material available at https://doi.org/10.1007/s00431-026-07507-z.

## Introduction

Down syndrome (DS), caused by a complete or partial extra copy of chromosome 21, is the most common chromosomal disorder and the leading genetic cause of intellectual disability [1–4]. Advances in paediatric care have substantially improved survival and life expectancy, increasing the need for long-term monitoring of developmental, functional and cardiometabolic health [3].

Cardiometabolic health is an increasing concern in this population. Children and adolescents with DS have a higher prevalence of overweight and obesity than typically developing peers, although BMI alone may not fully capture their metabolic vulnerability [6, 7]. Early abnormalities in glucose homoeostasis, lipid profile and liver function may occur even in the absence of severe excess weight [8]. Reduced physical activity, prolonged sedentary behaviour and low energy expenditure may further contribute to insulin resistance, dyslipidaemia, metabolic dysfunction-associated steatotic liver disease and increased cardiovascular risk [9–12].

Physical activity, sedentary behaviour and sleep represent potentially modifiable components of the 24-h behavioural profile [13–15]. International guidelines recommend at least 60 min per day of moderate-to-vigorous physical activity and limitation of sedentary time in children and adolescents [16]. However, individuals with DS face several biological, functional and contextual barriers that may limit their ability to meet these recommendations. Hypotonia, ligamentous laxity, delayed motor development, impaired balance and reduced muscle strength may restrict autonomy and participation in physical activity, while executive-function limitations, common comorbidities and restricted access to inclusive opportunities may further promote sedentary behaviour and reduced activity levels [1, 4, 5, 17–19].

Sleep is another key component of daily behaviour. Craniofacial narrowing, adenotonsillar hypertrophy, hypotonia and obesity predispose individuals with DS to sleep-disordered breathing and fragmented sleep [1–3]. Insufficient or disrupted sleep may impair daytime alertness, cognitive and behavioural functioning, participation in physical activity and metabolic regulation. Sleep duration, continuity, efficiency and regularity should therefore be considered together with physical activity and sedentary behaviour, as these domains interact throughout the day and may jointly influence cardiometabolic health [20–22].

Wearable-based assessment enables the objective and integrated characterization of these interrelated behaviours, including the stability and fragmentation of rest–activity rhythms under free-living conditions [23]. This approach is particularly valuable in DS, where intellectual disability may limit the reliability of self- or caregiver-reported measures. Accelerometer-derived data reduce recall bias and allow quantification of sedentary time and different activity intensities. Nevertheless, studies using objective measures to investigate 24-h behaviours and their potential health implications in paediatric DS remain limited and sometimes inconsistent [24, 25]. Beyond total activity and sleep duration, the temporal organization of these behaviours may provide complementary information about the 24-h behavioural profile. Bout duration and transitions between sedentary and active states characterize how movement is accumulated and interrupted, whereas rest–activity rhythm measures capture the timing, day-to-day stability and within-day fragmentation of activity and rest. In paediatric populations, prolonged sedentary accumulation and fewer interruptions have been associated with adiposity and cardiometabolic risk [26, 27], while experimental evidence suggests that interrupting prolonged sitting may improve glucose and insulin metabolism [28]. Multidimensional accelerometry-derived profiles incorporating movement volume and temporal characteristics have also been associated with differences in body composition, insulin sensitivity and metabolic risk beyond MVPA duration alone [29]. Similarly, greater fragmentation and altered timing of rest–activity rhythms have been associated with adiposity, lower cardiorespiratory fitness and selected cardiometabolic characteristics in children and adolescents [30, 31]. These measures may therefore complement conventional duration-based indicators when characterizing 24-h behaviour.

Therefore, this study aimed to characterize objectively measured 24-h movement behaviours in children and adolescents with DS, including physical activity, sedentary behaviour, sleep and rest–activity rhythmicity, and to compare their profiles with those of peers with normal weight and obesity. This multidimensional approach may help identify modifiable behavioural factors associated with cardiometabolic vulnerability and support the development of individualized preventive strategies.

## Patients and methods

### Study design

This cross-sectional study was performed as part of the “Fight against Pediatric Obesity: from a predictive tool for type 2 diabetes and cardiovascular disease risk to healthy educational programs” (PODiaCar) project. The protocol was approved by the Ethics Committee Lombardia 1 (approval number CET 202–2023). The study was conducted in accordance with the principles of the Declaration of Helsinki. Written informed consent was obtained from parents or legal guardians before participation. Assent was obtained from participants whenever appropriate according to age, developmental level and cognitive abilities. All data were anonymized and handled confidentially according to applicable data protection regulations.

#### Patients

This observational study included children and adolescents with DS consecutively referred through the ViviDown Association to the paediatric outpatient clinic of Buzzi Children’s Hospital in Milan, Italy, for auxological evaluation between January 2026 and May 2026. The diagnosis of DS was genetically confirmed in all participants by karyotype analysis or documented genetic testing. Participants were enrolled during routine clinical follow-up or a study-specific evaluation and were assessed according to a standardized protocol. Eligibility criteria for participants with DS were as follows: (1) genetically confirmed diagnosis of DS, (2) age between 8 and 18 years, (3) ability to walk independently or with minimal assistance and to wear the accelerometer during habitual daily activities, (4) clinical stability at the time of assessment and (5) availability of anthropometric data and valid accelerometer-derived physical activity, sleep and circadian-rhythm measures. Exclusion criteria were as follows: (1) acute illness, fever or infection at the time of assessment or during the accelerometer-monitoring period; (2) severe motor, neurological or orthopaedic impairment preventing habitual ambulation; (3) a medical contraindication to habitual physical activity assessment; (4) clinically unstable congenital or acquired cardiovascular disease; and (5) participation in a structured exercise, rehabilitation or lifestyle intervention specifically intended to modify physical activity during the assessment period. At enrolment, demographic and clinical information was collected from medical records and through interviews with parents or caregivers, and anthropometric measurements were obtained during the clinical assessment.

### Comparison groups

Two comparable groups of children and adolescents without DS were included for comparison: 17 participants with normal weight and 17 participants with obesity. Participants were selected from the same study framework and underwent the same standardized anthropometric and accelerometer-based assessment protocol as the DS group.

Weight status was classified using age- and sex-specific World Health Organization Body mass index (BMI)-for-age reference standards: normal weight was defined as a BMI z-score from − 1 to + 1 standard deviations, whereas obesity was defined as a BMI *z*-score greater than + 2 standard deviations [32].

The final analytical sample comprised 51 participants, with 17 individuals in each of the three groups: DS, normal weight and obesity.

## Methods

### Anthropometric assessment

Anthropometric measurements were performed by trained healthcare professionals using standardized procedures [8]. Body weight was measured with participants wearing light clothing and no shoes, using a calibrated electronic scale (Seca 864, Seca GmbH & Co., Hamburg, Germany), and recorded to the nearest 0.1 kg. Standing height was measured without shoes using a wall-mounted Harpenden stadiometer (Holtain Ltd., Crymych, UK) and recorded to the nearest 0.1 cm [8].

BMI was calculated as body weight in kg divided by height in metres squared. BMI *z*-score was calculated using appropriate age- and sex-specific reference standards [33]. Waist circumference was measured, when available, with a non-elastic tape at the midpoint between the lowest rib and the iliac crest, with the participant standing and breathing normally. Waist-to-height ratio was calculated as waist circumference divided by height [8].

### Physical activity and sedentary behaviour assessment

Physical activity, sleep and 24-h rest–activity rhythms were objectively assessed using a triaxial thigh-worn accelerometer (Fibion®, Fibion Inc., Jyväskylä, Finland). Participants were instructed to wear the device continuously for seven consecutive days while maintaining their usual daily activities, and participants and caregivers received standardized verbal and written instructions on device placement and use. Raw acceleration data were processed in R using the open-source GGIR package [34, 35], including autocalibration to local gravity, aggregation into 5-s epochs, calculation of the Euclidean Norm Minus One (ENMO, expressed in milli-gravitational units [mg]) and exclusion of non-wear periods identified from sustained low signal variability. Physical activity intensity was classified from ENMO using thresholds of < 28 mg for waking inactivity, 28 to < 270 mg for light physical activity, 270 to < 410 mg for moderate physical activity and ≥ 410 mg for vigorous physical activity; moderate-to-vigorous physical activity was defined as activity ≥ 270 mg [29]. Daily physical activity outcomes included time spent in waking inactivity; light, moderate and vigorous physical activity; moderate-to-vigorous physical activity calculated as the sum of moderate and vigorous activity; and mean acceleration as an indicator of overall movement intensity. The temporal organization of activity was further characterized by the mean duration of sedentary and physical activity bouts and by transition probabilities, indicating the likelihood of changing from sedentary time to physical activity or from physical activity to sedentary time. Sleep outcomes included sleep time, wake after sleep onset and sleep efficiency, calculated as the percentage of the sleep period spent asleep. Rest–activity rhythmicity was assessed using the Sleep Regularity Index, which indicates the consistency of sleep–wake states across consecutive days; interdaily stability, reflecting the reproducibility of the 24-h pattern between days; and intradaily variability, reflecting fragmentation and transitions between rest and activity within the day. L5 and M5 represented the mean acceleration during the least and most active consecutive 5-h periods, respectively, while their timing indicated when these periods occurred. Relative amplitude, calculated as $\left(M5-L5\right)/\left(M5+L5\right)$, quantified the contrast between the most and least active periods. Finally, a 24-h cosinor model was fitted to each participant’s activity profile to derive the mesor, amplitude, acrotime and model ${R}^{2}$, representing the rhythm-adjusted mean activity level, magnitude of rhythmic variation, estimated timing of peak activity and goodness of model fit, respectively.

### Statistical analysis

Continuous variables were summarized as mean and standard deviation and categorical variables as number and percentage. Group-specific outcome values were reported as arithmetic means with 95% confidence intervals in the original measurement units. Between-group differences in physical activity, activity-fragmentation, sleep and rest–activity rhythm outcomes were examined using analysis of covariance (ANCOVA), with group as the main factor and age and sex as covariates. Residual normality was assessed using the Shapiro–Wilk test; when residuals were not approximately normally distributed, the outcome was rank-transformed before fitting the same ANCOVA model. The adjusted overall group effect was evaluated using a partial *F* test and pairwise comparisons were performed using estimated marginal means with Tukey adjustment. To account for testing multiple outcomes, *p* values were adjusted using the Holm procedure separately within the physical activity volume, physical activity fragmentation, sleep and rest–activity rhythm domains. Analyses were conducted using complete observations available for each outcome. The overall 24-h behavioural profile was evaluated using permutational multivariate analysis of variance (PERMANOVA). Variables were standardized before calculating Euclidean distances. The PERMANOVA model included age, sex and group, with the marginal group effect evaluated using 9999 permutations constrained within the matched sets. Pairwise PERMANOVAs were adjusted using the Holm procedure. Homogeneity of multivariate dispersion was assessed using distances to the group median centroid and a permutation-based dispersion test. Principal component analysis was used to visualize differences in the overall behavioural profile. Each profile variable was first adjusted for age and sex using linear regression; the resulting residuals were standardized and entered into the principal component analysis. Component loadings were examined to identify the outcomes contributing most strongly to the first two principal components. Supplementary between-group analyses were expressed as age- and sex-adjusted standardized mean differences using Hedges g with 95% confidence intervals; positive values indicated higher values in the first-listed group. *p* values for these effect estimates were Holm-adjusted within each outcome domain and group comparison. As a sensitivity analysis, sleep and rest–activity rhythm models were additionally adjusted for the total number of valid circadian days and the proportion of valid days occurring on weekends, to assess whether differences in monitoring composition influenced the observed group effects. All statistical tests were two-sided, with statistical significance set at *p* < 0.05. Analyses were performed using R software (version 4.5.3; R Foundation for Statistical Computing, Vienna, Austria), using the *emmeans*, *vegan*, *permute* and *ggplot2* packages.

## Results

The descriptive characteristics of the sample divided by group are presented in Table 1.

**Table 1:** Descriptive characteristics of the total sample divided by the group

	Normal weight (*N* = 17)	Obesity (*N* = 17)	Down syndrome (*N* = 17)	Overall (*N* = 51)
Demographic and anthropometric characteristics				
Age, years	10.4 (0.9)	11.6 (2.4)	14.1 (2.8)	12.0 (2.6)
Sex				
Male, *n* (%)	13 (76.5%)	13 (76.5%)	13 (76.5%)	39 (76.5%)
Female, *n* (%)	4 (23.5%)	4 (23.5%)	4 (23.5%)	12 (23.5%)
Weight, kg	31.4 (3.5)	68.0 (17.7)	49.2 (11.1)	49.5 (19.3)
Height, cm	138.9 (6.9)	152.4 (11.9)	146.5 (11.1)	145.9 (11.5)
Waist circumference, cm	60.2 (2.8)	88.7 (10.8)	72.0 (8.5)	73.8 (14.7)
BMI *z*-score	− 0.3 (0.6)	3.1 (0.8)	1.1 (1.2)	1.3 (1.7)
Waist-to-height ratio	0.4 (0.0)	0.6 (0.1)	0.5 (0.1)	0.5 (0.1)
Physical activity				
Sedentary time, min/day	435.5 (131.5)	770.4 (94.2)	742.9 (75.1)	649.6 (183.5)
Light PA, min/day	428.3 (106.3)	174.6 (56.1)	168.0 (52.2)	257.0 (143.1)
Moderate PA, min/day	58.8 (32.1)	17.6 (11.7)	14.2 (7.4)	30.2 (28.5)
Vigorous PA, min/day	4.0 (5.0)	4.7 (4.2)	4.8 (4.1)	4.5 (4.3)
MVPA, min/day	62.9 (34.9)	22.3 (13.8)	18.9 (10.9)	34.7 (29.9)
Sleep				
Sleep, min/night	493.2 (54.3)	469.1 (59.9)	503.9 (49.3)	488.7 (55.6)
Sleep efficiency, (%)	72.1 (8.4)	86.0 (4.6)	73.5 (8.4)	77.2 (9.6)
Sleep regularity index, (%)	82.9 (9.5)	83.7 (7.8)	75.3 (13.6)	80.6 (11.1)
Circadian rhythm				
Interdaily stability	0.7 (0.1)	0.5 (0.1)	0.5 (0.1)	0.5 (0.1)
Intradaily variability	0.6 (0.1)	1.0 (0.3)	1.0 (0.2)	0.9 (0.3)
L5 activity level, mg	9.1 (5.3)	2.7 (2.3)	3.7 (1.4)	5.2 (4.4)
M5 activity level, mg	105.9 (25.9)	52.3 (19.1)	47.0 (13.3)	68.4 (33.3)
Relative amplitude	0.8 (0.1)	0.9 (0.1)	0.8 (0.1)	0.9 (0.1)
Cosinor mesor	3.0 (0.3)	2.0 (0.3)	2.0 (0.2)	2.4 (0.5)
Cosinor amplitude	1.1 (0.1)	1.0 (0.3)	0.9 (0.3)	1.0 (0.3)

Data are presented as mean (standard deviation) unless otherwise indicated. Categorical variables are reported as number (percentage). *BMI* body mass index, *PA* physical activity, *MVPA* moderate-to-vigorous physical activity, *L5* mean activity level during the least active consecutive 5-h period, *M5* mean activity level during the most active consecutive 5-h period, *mg* milli-gravitational units

**Fig. 1:**
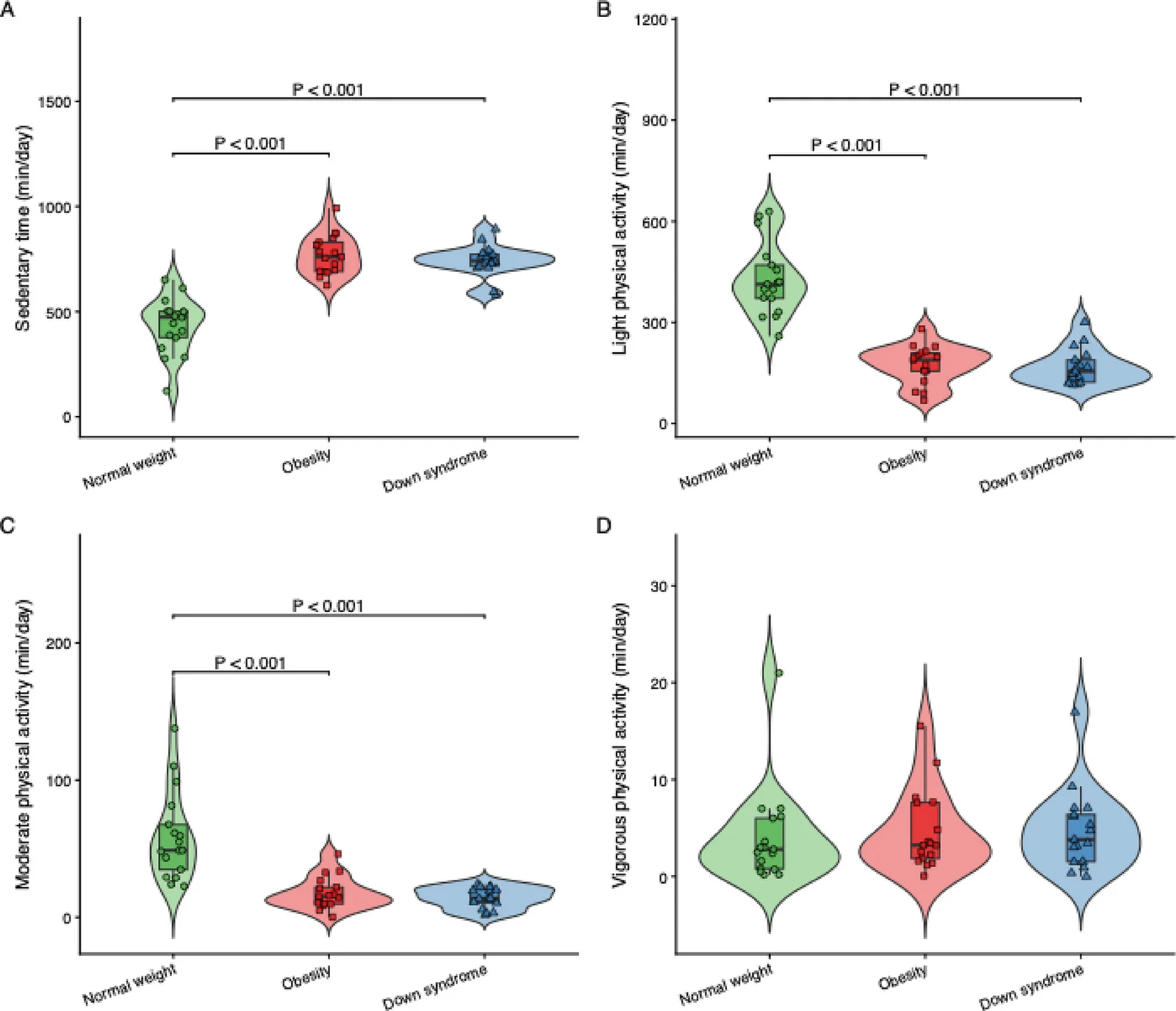
Physical activity outcomes by group. Violin plots show the outcome distributions, individual observations and embedded boxplots. *p* values indicate significant Tukey-adjusted pairwise comparisons from age- and sex-adjusted ANCOVA models; non-significant comparisons are not displayed

Age- and sex-adjusted analyses showed marked between-group differences across most physical activity outcomes (Table 2 and Fig. 1). The normal-weight group accumulated substantially less sedentary time than the obesity and DS groups (both *p* < 0.001). Conversely, the normal-weight group accumulated more light physical activity (428.3 min/day [373.6–482.9]), moderate physical activity (58.8 min/day [42.3–75.3]) and moderate-to-vigorous physical activity (62.9 min/day [44.9–80.8]) than both the obesity and DS groups (all *p* < 0.001). Vigorous physical activity did not differ among groups, and no physical activity volume outcome differed significantly between the obesity and DS groups. The temporal accumulation of activity also differed among groups. Relative to both the obesity and DS groups, the normal-weight group had shorter sedentary bouts, longer physical-activity bouts, a higher probability of transitioning from sedentary time to physical activity and a lower probability of transitioning from physical activity to sedentary time. Mean sedentary-bout duration was 7.35 min (95% CI, 6.17–8.52) in the normal-weight group, compared with 53.46 min (18.80–88.12) in the obesity group and 37.17 min (29.77–44.58) in the DS group (both pairwise *p* < 0.001). The corresponding probability of transitioning from sedentary time to physical activity was 0.145 (0.111–0.180), 0.032 (0.023–0.042) and 0.031 (0.023–0.039), respectively (both comparisons with the normal-weight group *p* < 0.001).

**Table 2:** Comparison of physical activity, sleep and circadian outcomes among groups

	Normal weight mean (95% CI)	Obesity mean (95% CI)	Down syndrome mean (95% CI)	Normal weight vs obesity	Normal weight vs Down syndrome	Obesity vs Down syndrome
Physical activity volume						
Sedentary time, min/day	435.5 (367.9 to 503.1)	770.4 (721.9 to 818.8)	742.9 (704.3 to 781.5)	*p* < 0.001	*p* < 0.001	*p* > 0.05
Light physical activity, min/day	428.3 (373.6 to 482.9)	174.6 (145.7 to 203.4)	168.0 (141.2 to 194.9)	*p* < 0.001	*p* < 0.001	*p* > 0.05
Moderate physical activity, min/day	58.8 (42.3 to 75.3)	17.6 (11.6 to 23.6)	14.2 (10.4 to 18.0)	*p* < 0.001	*p* < 0.001	*p* > 0.05
Vigorous physical activity, min/day	4.0 (1.5 to 6.6)	4.7 (2.6 to 6.8)	4.8 (2.6 to 6.9)	*p* > 0.05	*p* > 0.05	*p* > 0.05
Moderate-to-vigorous physical activity, min/day	62.9 (44.9 to 80.8)	22.3 (15.2 to 29.4)	18.9 (13.3 to 24.5)	*p* < 0.001	*p* < 0.001	*p* > 0.05
Mean acceleration, mg	70.9 (60.3 to 81.5)	31.0 (25.8 to 36.2)	29.2 (25.3 to 33.0)	*p* < 0.001	*p* < 0.001	*p* > 0.05
Physical activity fragmentation						
Mean duration of sedentary bouts, min	7.35 (6.17 to 8.52)	53.46 (18.80 to 88.12)	37.17 (29.77 to 44.58)	*p* < 0.001	*p* < 0.001	*p* > 0.05
Mean duration of physical-activity bouts, min	10.06 (7.35 to 12.76)	6.50 (5.73 to 7.28)	6.62 (5.58 to 7.66)	*p* = 0.002	*p* = 0.037	*p* > 0.05
Transition probability: sedentary time to physical activity	0.145 (0.111 to 0.180)	0.032 (0.023 to 0.042)	0.031 (0.023 to 0.039)	*p* < 0.001	*p* < 0.001	*p* > 0.05
Transition probability: physical activity to sedentary time	0.112 (0.096 to 0.128)	0.161 (0.143 to 0.180)	0.163 (0.140 to 0.186)	*p* = 0.003	*p* = 0.027	*p* > 0.05
Sleep						
Sleep time, min/night	493.2 (465.2 to 521.1)	469.1 (438.3 to 499.9)	503.9 (478.6 to 529.3)	*p* > 0.05	*p* > 0.05	*p* = 0.011
Wake after sleep onset, min/night	127.6 (104.3 to 150.9)	64.7 (53.3 to 76.1)	134.1 (110.6 to 157.5)	*p* < 0.001	*p* > 0.05	*p* < 0.001
Sleep efficiency, %	72.1 (67.8 to 76.4)	86.0 (83.6 to 88.3)	73.5 (69.2 to 77.9)	*p* < 0.001	*p* > 0.05	*p* < 0.001
Circadian rhythm						
Sleep Regularity Index, %	82.9 (78.0 to 87.8)	83.7 (79.7 to 87.7)	75.3 (68.3 to 82.3)	*p* > 0.05	*p* > 0.05	*p* > 0.05
Interdaily stability	0.663 (0.607 to 0.718)	0.482 (0.441 to 0.524)	0.487 (0.428 to 0.546)	*p* < 0.001	*p* = 0.030	*p* > 0.05
Intradaily variability	0.621 (0.544 to 0.697)	1.015 (0.877 to 1.154)	1.003 (0.878 to 1.128)	*p* < 0.001	*p* = 0.008	*p* > 0.05
L5 activity, mg	9.1 (6.4 to 11.8)	2.7 (1.5 to 3.9)	3.7 (3.0 to 4.4)	*p* < 0.001	*p* < 0.001	*p* > 0.05
M5 activity, mg	105.9 (92.6 to 119.2)	52.3 (42.5 to 62.1)	47.0 (40.2 to 53.9)	*p* < 0.001	*p* < 0.001	*p* > 0.05
L5 timing, clock hour	24.20 (23.56 to 24.83)	26.64 (25.92 to 27.36)	25.11 (24.65 to 25.57)	*p* < 0.001	*p* > 0.05	*p* = 0.003
M5 timing, clock hour	11.57 (10.86 to 12.29)	15.07 (14.11 to 16.02)	13.56 (12.96 to 14.16)	*p* < 0.001	*p* = 0.015	*p* = 0.033
Relative amplitude	0.841 (0.806 to 0.875)	0.896 (0.860 to 0.933)	0.847 (0.813 to 0.881)	*p* < 0.001	*p* > 0.05	*p* > 0.05
Cosinor mesor	3.01 (2.88 to 3.13)	2.01 (1.88 to 2.14)	2.04 (1.96 to 2.12)	*p* < 0.001	*p* < 0.001	*p* > 0.05
Cosinor amplitude	1.137 (1.070 to 1.205)	0.985 (0.833 to 1.137)	0.870 (0.701 to 1.038)	*p* > 0.05	*p* > 0.05	*p* > 0.05
Cosinor acrotime, clock hour	13.67 (13.12 to 14.22)	16.98 (16.29 to 17.66)	15.74 (15.36 to 16.12)	*p* < 0.001	*p* < 0.001	*p* = 0.006
Cosinor model *R*^2^	0.350 (0.314 to 0.387)	0.253 (0.196 to 0.310)	0.214 (0.147 to 0.282)	*p* > 0.05	*p* > 0.05	*p* > 0.05

Values are arithmetic means (95% confidence intervals) in the original measurement units. When residuals were not approximately normal, rank-transformed ANCOVA was used. Pairwise comparisons were adjusted using Tukey’s method

**Fig. 2:**
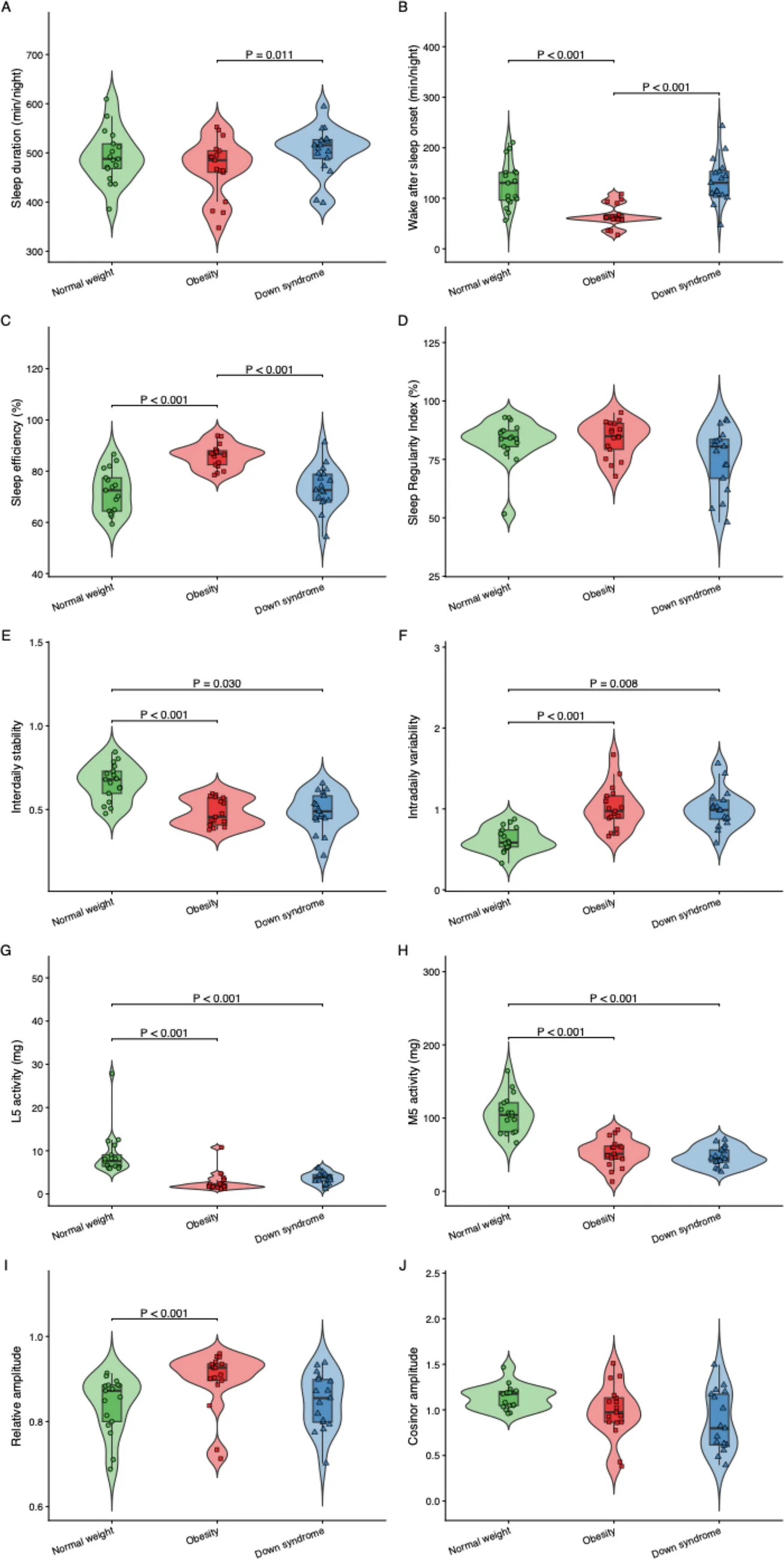
Sleep and circadian-rhythm outcomes by group. Violin plots show the outcome distributions, individual observations and embedded boxplots. *p* values indicate significant Tukey-adjusted pairwise comparisons from age- and sex-adjusted ANCOVA models; non-significant comparisons are not displayed

Sleep outcomes displayed a different pattern of between-group variation (Table 2 and Fig. 2). Sleep duration differed significantly among groups (global *p* = 0.015), with participants with DS showing longer sleep duration than participants with obesity (503.9 min/night [95% CI, 478.6–529.3] vs 469.1 min/night [438.3–499.9]; Tukey-adjusted *p* = 0.011). Sleep duration in the normal-weight group (493.2 min/night [465.2–521.1]) did not differ significantly from either of the other groups. Participants with obesity had lower wake after sleep onset than both the normal-weight and DS groups (64.7 min/night [95% CI, 53.3–76.1] vs 127.6 [104.3–150.9] and 134.1 [110.6–157.5], respectively; both *p* < 0.001). Participants with obesity also showed higher sleep efficiency (86.0% [83.6–88.3]) than the normal-weight (72.1% [67.8–76.4]) and DS groups (73.5% [69.2–77.9]; both *p* < 0.001).

M5 timing and cosinor acrotime showed the same ordering, whereas L5 timing was later in the obesity group than in both the normal-weight and DS groups. The normal-weight group also showed greater interdaily stability and lower intradaily variability than the obesity and DS groups. Interdaily stability was 0.663 (95% CI, 0.607–0.718), 0.482 (0.441–0.524) and 0.487 (0.428–0.546), respectively, whereas intradaily variability was 0.621 (0.544–0.697), 1.015 (0.877–1.154) and 1.003 (0.878–1.128), respectively. L5 activity, M5 activity and cosinor mesor were likewise higher in the normal-weight group than in both comparison groups. Relative amplitude was higher in the obesity group than in the normal-weight group, while the Sleep Regularity Index, cosinor amplitude and cosinor model *R*-squared did not differ significantly among groups. All participants contributed at least one valid weekend day, with a median of two valid weekend days in each group. In sensitivity analyses additionally accounting for the total number of valid circadian days and the proportion of weekend days, the main sleep and rest–activity findings were largely unchanged (Supplementary Table S1). Group effects remained significant for sleep duration, wake after sleep onset, sleep efficiency, intradaily variability, L5 and M5 activity and timing, cosinor mesor and cosinor acrotime. In contrast, the group effects for interdaily stability and relative amplitude were attenuated and were no longer significant after Holm correction.

The overall 24-h behavioural profile differed among groups in the matched PERMANOVA analysis (*R*-squared = 0.296, *p* < 0.001), with no evidence of unequal multivariate dispersion (*p* = 0.524; Supplementary Figure S1). The first two principal components explained 62.8% of the total variance (PC1, 47.2%; PC2, 15.6%). Visual separation occurred primarily along PC1, with normal-weight participants generally displaying higher PC1 scores and participants with obesity or DS showing lower and largely overlapping scores. PC1 contrasted greater sedentary time and intradaily variability with a higher probability of transitioning from inactivity to physical activity, greater light and moderate-to-vigorous physical activity, higher M5 activity, greater interdaily stability and longer physical-activity bouts. Adjusted standardized between-group effects are provided in Supplementary Figures S2-S4 and were broadly consistent with the outcome-specific analyses: differences were extensive between the normal-weight group and both comparison groups, whereas the obesity and DS groups differed primarily in sleep.

## Discussion

This study objectively characterized 24-h movement behaviours in children and adolescents with DS, compared with peers with normal weight and obesity. Participants with DS showed more sedentary time, lower light and moderate-to-vigorous physical activity, greater activity fragmentation and less stable rest–activity rhythms than normal-weight peers. Their overall profile largely overlapped with that of participants with obesity. Sleep duration was not reduced, but sleep was more fragmented and less efficient than in the obesity group.

These findings can first be interpreted in light of the multiple barriers to habitual movement that affect individuals with DS [17, 36]. Hypotonia, reduced muscle strength, impaired balance, ligamentous laxity and delayed motor development may increase the effort required for daily activities and limit participation in active play and structured exercise [17, 36]. Executive-function difficulties, family concerns, limited access to inclusive programmes and environmental barriers may further reinforce sedentary routines [17]. The observed profile is therefore likely to reflect the combined influence of biological, functional and contextual factors.

Against this background, a particularly relevant finding was the similarity between the DS and obesity groups across most physical activity outcomes. Both accumulated substantially more sedentary time and less light and moderate-to-vigorous physical activity than normal-weight peers. However, although BMI interpretation in individuals with DS may be affected by their characteristic short stature and altered body proportions [37], the substantially lower mean BMI *z*-score observed in the DS group suggests that the low-movement phenotype cannot be explained solely by excess adiposity. Rather, DS-related motor and developmental limitations may lead to activity patterns resembling those associated with obesity, even before marked weight excess develops. Conversely, prolonged inactivity may contribute to progressive adiposity and metabolic impairment, reinforcing a bidirectional relationship between functional limitations and cardiometabolic risk [38, 39].

Beyond total activity volume, the organization of movement across the day also differed markedly. Participants with DS and obesity had longer sedentary bouts, shorter periods of sustained activity and a lower probability of transitioning from inactivity to movement. These findings indicate that total activity minutes alone do not fully capture daily behaviour [40]. The pattern in which sedentary time is accumulated may also be relevant, as prolonged uninterrupted sedentary periods and fewer transitions to activity have been associated with adiposity and cardiometabolic risk in paediatric populations [26, 27]. Experimental evidence further suggests that regularly interrupting prolonged sitting may improve glucose and insulin responses in children with overweight or obesity [28]. However, as cardiometabolic outcomes were not examined in the present study, the clinical consequences of the observed bout patterns in youth with DS remain to be established. These findings suggest that, alongside increasing overall physical activity, reducing prolonged uninterrupted sedentary periods may represent a relevant target for future intervention studies in youth with DS. [16, 41].

This fragmented pattern also has practical implications for intervention targets. Only the normal-weight group approached the recommended average of at least 60 min per day of moderate-to-vigorous physical activity, whereas the DS and obesity groups accumulated approximately 20 min per day. Vigorous activity was low in all groups. For children and adolescents with DS, progressive increases in light and moderate activity may therefore be more realistic than an exclusive focus on vigorous exercise [24, 42]. Walking, adapted sports, structured play, dancing, active transport and strength or balance activities may provide sustainable benefits.

In addition to reduced activity volume and greater fragmentation, the rest–activity findings revealed differences in the temporal organization of behaviour. Participants with DS showed lower day-to-day stability, greater within-day fragmentation, lower activity during the most active 5-h period and a later peak than normal-weight peers. Similar alterations in the obesity group suggest a shared pattern of reduced and less consistently organized daytime movement. Most rest–activity differences were robust to adjustment for weekday/weekend monitoring composition, although the group effects for interdaily stability and relative amplitude were attenuated. These findings suggest that some rhythm measures may be particularly sensitive to the temporal composition of the monitoring period, while the broader pattern of altered rest–activity organization remained evident.

The delayed timing of peak activity further complements this pattern of weaker rest–activity organization. More regular and earlier daytime movement may reinforce circadian organization and promote sleep pressure at night, whereas low daytime activity and delayed peaks may contribute to weaker rest–activity rhythms [43, 44]. Although causal relationships cannot be established, these findings support interventions that combine greater daytime movement with more consistent schedules and regular wake times.

These circadian findings should be considered alongside sleep duration and continuity. Participants with DS slept longer than those with obesity but experienced greater wake after sleep onset and lower sleep efficiency, indicating that adequate sleep duration did not necessarily translate into restorative sleep [45]. This finding is clinically plausible given the high predisposition of children with DS to sleep-disordered breathing, related to craniofacial narrowing, adenotonsillar hypertrophy, hypotonia and upper-airway abnormalities [3]. Sleep fragmentation may adversely affect daytime alertness, behaviour, learning, appetite regulation and engagement in physical activity [46]. Wearable-derived sleep measures should therefore be integrated with clinical screening for snoring, witnessed apnoeas, restless sleep and daytime symptoms, while polysomnography remains essential when sleep-disordered breathing is suspected.

Physical activity, sedentary behaviour and sleep are interdependent components of the same 24-h period. Fragmented sleep may reduce daytime energy and movement, while prolonged sedentary behaviour and insufficient daytime activity may weaken circadian organization and sleep quality [22]. Together, these behaviours may adversely influence insulin sensitivity, appetite regulation, adiposity, blood pressure and lipid metabolism [20–22]. This interaction is especially relevant in DS, where metabolic and hepatic abnormalities may occur without marked obesity [8]. An integrated assessment may therefore identify risk more effectively than BMI or isolated activity measures alone.

Taken together, the overlap between the DS and obesity groups has important clinical implications. Children with DS may present an unfavourable behavioural profile comparable with that of peers with obesity even when they do not meet conventional obesity criteria. Routine follow-up should therefore include assessment of physical activity, sedentary accumulation, sleep quality and daily regularity alongside anthropometric and metabolic evaluation. Early recognition of adverse patterns may allow intervention before excess weight or biochemical abnormalities develop.

Interventions should be individualized, accessible and family-centred. Practical goals may include replacing sedentary time with light activity, introducing regular movement breaks, gradually increasing moderate activity and improving daily routines. Programmes integrated into school, home and community settings may be more sustainable than isolated exercise sessions. Tele-coaching and hybrid models may further reduce access barriers and support caregivers [47].

Within this integrated approach, accelerometers and other wearable devices are particularly useful because they reduce reliance on self-report and provide objective information on the intensity, timing, duration and fragmentation of movement and sleep [48, 49]. They may help identify behavioural targets, monitor adherence and evaluate responses to lifestyle or rehabilitation programmes. Repeated assessments could support personalized recommendations, such as increasing morning movement, shortening sedentary bouts or improving day-to-day consistency.

However, wearable-derived measures require careful interpretation [50, 51]. Estimates may vary according to device placement, analytical algorithms, activity thresholds and non-wear definitions [52]. Sleep algorithms cannot diagnose sleep-disordered breathing or characterize sleep architecture. Wearables should therefore be integrated with clinical assessment and, when appropriate, cardiometabolic testing and formal sleep evaluation.

This study has several limitations. Its cross-sectional design precludes causal inference, while the relatively small sample size limited the possibility of conducting subgroup analyses according to sex, pubertal stage, degree of intellectual disability, weight status within the DS group and associated comorbidities. Information on thyroid function, sleep apnoea, medication use, school schedules and environmental determinants was not included. Accelerometry also cannot assess respiratory abnormalities or sleep architecture. Finally, the inclusion of ambulatory and clinically stable participants may limit generalizability to individuals with more severe impairment.

Future longitudinal studies should determine whether 24-h behavioural profiles predict changes in adiposity, glucose metabolism, liver health and cardiovascular risk in children with DS. Larger samples would allow identification of distinct behavioural phenotypes and evaluation of the contribution of motor function, comorbidities and social determinants. Combining wearable data with polysomnography, cardiometabolic biomarkers and dietary assessment may clarify the mechanisms linking daily behaviours to health outcomes. Intervention studies should also establish whether modifying the timing and fragmentation of movement and sleep provides benefits beyond increasing total activity.

In conclusion, children and adolescents with DS showed a highly sedentary, low-movement and less stable 24-h behavioural profile that closely resembled that of peers with obesity and differed substantially from normal-weight peers. Sleep duration was not consistently reduced, but nocturnal fragmentation remained clinically relevant. These findings support an integrated preventive approach in which physical activity, sedentary behaviour, sleep quality and daily rhythmicity are assessed together. Objective monitoring through accelerometers and wearable devices may facilitate early risk identification, individualized counselling and longitudinal follow-up, supporting strategies to improve functional health and reduce cardiometabolic vulnerability.

## Supplementary information

Below is the link to the electronic supplementary material.

ESM 1 (PDF 95.6 KB)

ESM 2 (PDF 267 KB)

ESM 3 (PDF 312 KB)

ESM 4 (PDF 314 KB)

ESM 5 (PDF 311 KB)

## Data Availability

Data are available on request from the authors.
