# Supplementary material for: Accelerometer-based 24-h movement behaviours in children and adolescents with Down syndrome
Source: Eur J Pediatr. 2026 Oct 12;185(11):833. doi: 10.1007/s00431-026-07507-z (PMC13658301; doi:10.1007/s00431-026-07507-z)
Supplement: Supplementary file 1 [file 431_2026_7507_MOESM1_ESM.pdf]

Supplementary Table S1. Comparison of primary and weekend-coverage–adjusted group effects

Domain	Outcome	N	Primary F	Primary partial η²	Primary Holm-adjusted p	Weekend-adjusted F	Weekend-adjusted partial η²	Weekend-adjusted Holm-adjusted p
**Sleep**	Sleep duration	51	4.64	0.168	0.015	5.23	0.192	0.009
	Wake after sleep onset	51	15.92	0.409	<0.001	8.16	0.271	0.003
	Sleep efficiency	51	18.12	0.441	<0.001	6.68	0.233	0.006
	Sleep Regularity Index	51	1.05	0.044	0.719	1.49	0.063	0.496
**Circadian rhythm**	Interdaily stability	51	10.91	0.322	<0.001	4.32	0.164	0.097
	Intradaily variability	51	11.20	0.327	<0.001	7.95	0.265	0.007
	L5 activity	51	47.22	0.672	<0.001	22.92	0.510	<0.001
	M5 activity	51	30.30	0.569	<0.001	21.06	0.489	<0.001
	L5 timing	51	17.41	0.431	<0.001	9.79	0.308	0.002
	M5 timing	51	20.93	0.476	<0.001	9.36	0.298	0.003
	Relative amplitude	51	7.86	0.255	0.005	2.19	0.091	0.496
	Cosinor mesor	51	35.75	0.609	<0.001	25.91	0.541	<0.001
	Cosinor amplitude	51	0.49	0.021	0.719	1.21	0.052	0.496
	Cosinor acrotime	51	38.91	0.628	<0.001	16.36	0.427	<0.001
	Cosinor model R-squared	51	2.06	0.082	0.415	2.15	0.089	0.496

Notes: Both models weere adjusted for age and sex. The sensitivity model additionally adjusted for the total number of valid circadian days and the proportion of valid weekend days. Global group p values were Holm-adjusted within the sleep and circadian-rhythm domains. F values and partial η² refer to the adjusted global group effect. All participants contributed at least one valid weekend day.
