# Supplementary material for: Accelerometer-based 24-h movement behaviours in children and adolescents with Down syndrome
Source: Eur J Pediatr. 2026 Oct 12;185(11):833. doi: 10.1007/s00431-026-07507-z (PMC13658301; doi:10.1007/s00431-026-07507-z)
Supplement: Supplementary file 2 [file 431_2026_7507_MOESM2_ESM.pdf]

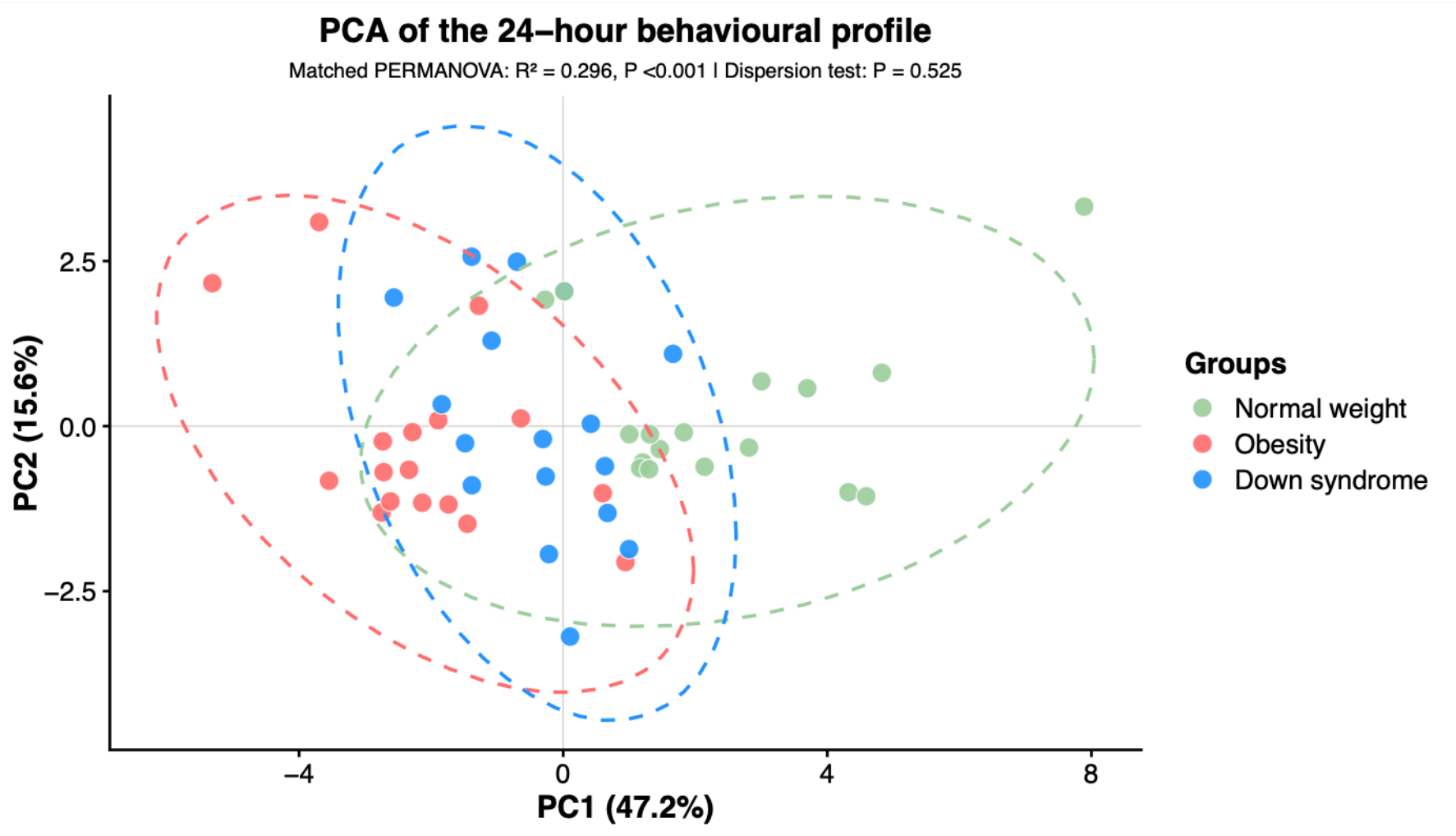

PC1 feature	Loading
Sedentary time	−0.391
Inactivity–to–PA transition	0.378
Light PA	0.371
M5 activity	0.354
MVPA	0.320
Intradaily variability	−0.282
Interdaily stability	0.279
PA–bout duration	0.263

PC2 feature	Loading
Relative amplitude	−0.542
Sleep regularity	−0.494
PA–bout duration	0.329
Intradaily variability	0.324
Sleep duration	−0.305
Sleep efficiency	−0.256
Inactivity–bout duration	0.232
Interdaily stability	−0.145

Supplementary Figure S1. Principal component analysis of the 24-hour behavioural profile. Points represent participants and dashed ellipses summarise the distributions of the three groups.
