# Supplementary material for: Accelerometer-based 24-h movement behaviours in children and adolescents with Down syndrome
Source: Eur J Pediatr. 2026 Oct 12;185(11):833. doi: 10.1007/s00431-026-07507-z (PMC13658301; doi:10.1007/s00431-026-07507-z)
Supplement: Supplementary file 5 [file 431_2026_7507_MOESM5_ESM.pdf]

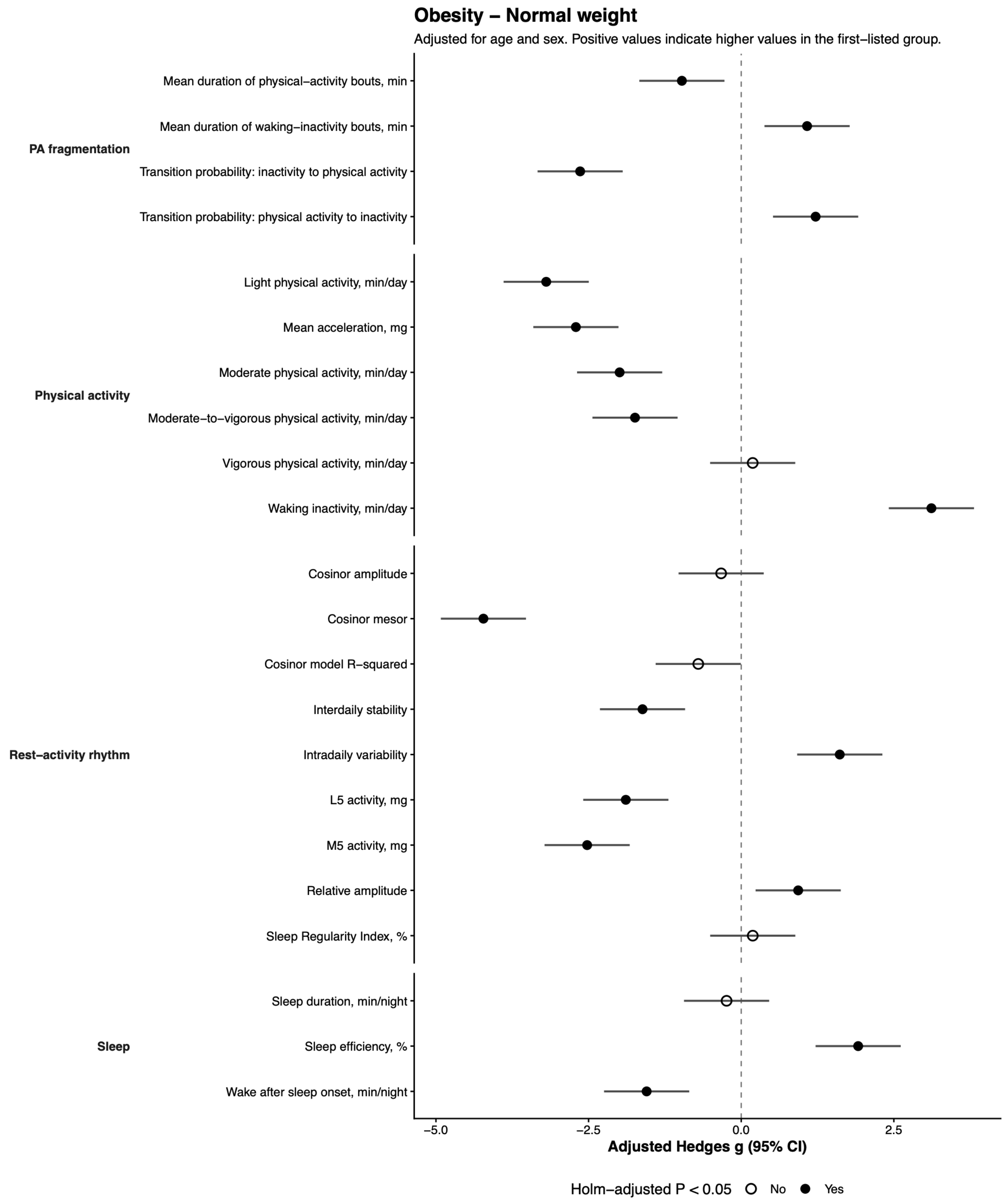

Supplementary Figure S4. Adjusted standardized mean differences for Obesity versus normal weight. Estimates are adjusted for age and sex and are presented as Hedges g with 95% confidence intervals. Positive values indicate higher values in the first-listed group. Filled circles indicate Holm-adjusted $P<0.05$.
